# Hemi-Clamshell Approach for Fetal Lung Interstitial Tumor Resection in a Neonate: A Case Report

**DOI:** 10.1055/s-0041-1735807

**Published:** 2021-11-24

**Authors:** Yasuhiro Kuroda, Hiroaki Fukuzawa, Insu Kawahara, Keiichi Morita

**Affiliations:** 1 Department of Pediatric Surgery, Kobe Children's Hospital, Chuo-ku, Kobe, Japan

**Keywords:** fetal lung interstitial tumor, hemi-clamshell approach, neonate tumors

## Abstract

Fetal lung interstitial tumor (FLIT) is a rare primary lung mass in neonates. Classical incisions, such as posterolateral thoracotomy or median sternotomy, do not provide optimal exposure of the operative field for the resection of pediatric thoracic giant tumors. Herein, we report a rare case of a FLIT in a full-term male neonate, with complete resection achieved using a hemi-clamshell approach, which provided the required visualization of the operative field. The neonate was transferred to our hospital because of mild respiratory distress, which developed 18-hour after normal vaginal delivery. A mass in his right chest, without a midline shift, was observed on chest radiographs. Computed tomography showed a well-circumscribed solid anterior cervicothoracic mass, with a uniform density and no apparent cysts, diagnosed as a primary thoracic giant tumor. Once the patient was clinically stabilized, we proceeded with right upper lobectomy, using a hemi-clamshell approach, full sternotomy, and anterolateral thoracotomy, on postnatal day 22.

Histopathologic examination revealed an 8.5 × 6.5 × 4.0 cm solid mass within the right upper lobe, which was diagnosed as a FLIT. His postoperative recovery was uneventful. The patient was followed up for 1 year, with no complaints or symptoms and no postoperative shoulder dysfunction. Gross total resection of primary thoracic giant tumors can be accomplished in neonates with optimal exposure of the chest cavity using a hemi-clamshell approach.

## Introduction

For thoracic giant tumors in pediatric patients, isolated posterolateral thoracotomy and median sternotomy incision approach provide insufficient exposure to achieve complete resection. 1 However, a hemi-clamshell incision, consisting of a median sternotomy combined with an anterolateral thoracotomy, 2 does provide optimal exposure of the hilar and mediastinal vascular structures, although its use in neonates has not been previously reported. Herein, we describe a rare case of resection of a fetal lung interstitial tumor (FLIT) in a 22-day-old neonate using a hemi-clamshell access.

## Case Presentation

The patient was a male neonate (gestation, 40 weeks; birth weight, 3.400 g; APGAR score at 1 and 5 minutes, 8 and 9, respectively). He developed tachypnea and mild respiratory distress 18 hour after delivery. A chest radiograph showed a mass in the right chest without a midline shift to the left, which had not been identified on prenatal routine ultrasound. The neonate was transferred to our neonatal intensive care unit for assessment and treatment at Christmas time.

**Fig. 1:**
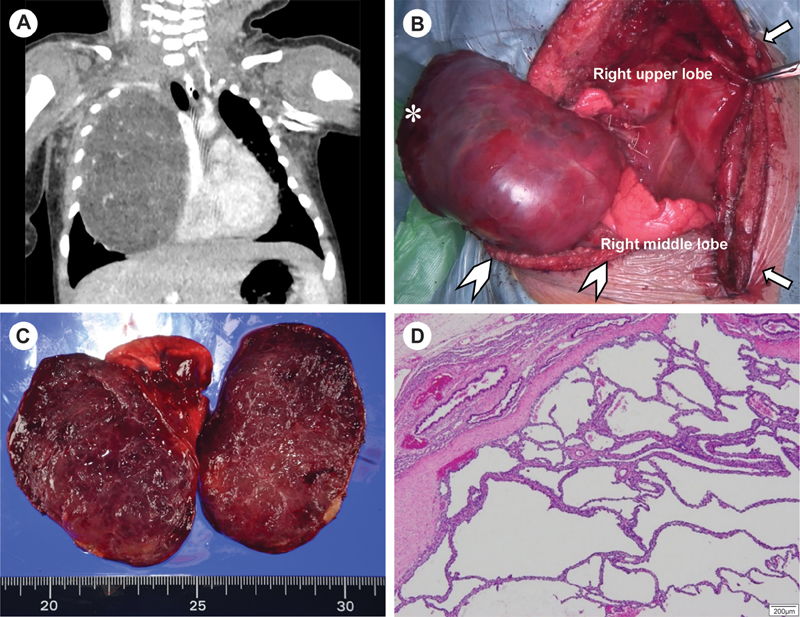
( **A** ) Coronal computed tomography image of the fetal lung interstitial tumor. The image was obtained on postnatal day 6, showing a homogeneous, enhanced, well-demarcated intrapulmonary mass (8.5 cm in diameter) with no apparent systemic vascularization and no infiltration into adjacent structures. ( **B** ) The hemi-clamshell incision consists of full sternotomy (arrow) combined with an anterolateral thoracotomy (arrowhead), through the fifth intercostal space. The giant tumor was continuous with the right upper lobe and adhered moderately to the surround tissues at the cervicothoracic junction (asterisk). ( **C** ) Macroscopic findings: Histopathological examination revealed a well-circumscribed (8.5 × 6.5 × 4.0 cm) solid mass within the right upper lobe, with a solid and spongy texture and a homogeneous surface and diffuse microcysts. ( **D** ) Pathological findings: Histologic image of the fetal lung interstitial tumor showing a well-circumscribed tumorous lesion, with a fibrous border and an abrupt interface with the adjacent normal lung.

On admission, he was hemodynamically stable but cyanotic with mild respiratory acidosis. Compression on the right lung by the mass was suspected; he underwent nasal directional positive airway pressure ( *n* -DPAP), which improved his condition. All serum levels were within the normal range. The patient's alpha fetoprotein (65,223.6 ng/mL), human chorionic gonadotropin (<0.1 ng/mL), urinary vanillylmandelic acid (6.4 µg/mg Cr), homovanillic acid (13.2 µg/mg Cr), and neuron-specific enolase (35.1 ng/mL) levels were not abnormal. Computed tomography (CT) imaging under sedation used midazolam, and *n* -DPAP showed a well-circumscribed solid anterior cervicothoracic mass of uniform density, 6.6 × 4.6 × 4.8 cm in size, and with no apparent cysts ( Fig. 1A ). It was suspected that arterial vessels from the pulmonary artery were supplied to the tumor. It was uncertain at that time if this was a mediastinal or a lung tumor. As recommended by our pediatric tumor board and a pediatric radiology department, magnetic resonance imaging did not show fat, fluid, and calcified components. On ultrasonography, the tumor was asynchronous with the heartbeat; moreover, the thymus presents normal signal intensity, and the border between the thymus and the tumor was clear. We thought he had the tumor from the fetal period. All things considered, we suspected a primary right lung tumor, not a mediastinal tumor. Type 3 congenital pulmonary airway malformation (CPAM), infantile fibrosarcoma, type 3 pleuropulmonary blastoma, and FLIT were differential diagnoses. Considering the possibility of low efficacy of chemotherapy and major bleeding, we thought that complete surgical resection was more important than the biopsy in his uncertain tumor. After clinical stabilization and waiting for the hospital to open after the New Year holidays, the neonate was taken to the operating theater on day 22 of life. The hemi-clamshell approach was performed with cardiac surgery as backup to safely control and dissect the hilar pulmonary vessels.

Based on prior knowledge and experience, we knew that a posterolateral thoracotomy or median sternotomy would not provide the necessary exposure of the operative field for such a large tumor of the anterior cervicothoracic junction. The patient was positioned in the supine position with the arm slightly abducted. A full median sternotomy was initially performed to avoid vessel injuries and spillage for the complete resection of the tumor and provide optimal exposure of the hilar and mediastinal vascular structures before employing right thoracotomy with the hemi-clamshell approach. No adhesions were observed under the sternum. We proceeded with a hemi-clamshell approach, clipping the right internal thoracic artery and entering through the fifth intercostal space. The giant tumor (8.5 × 6.5 × 4.0 cm) was observed to be continuous with the right upper lobe (5.0 × 2.5 × 2.0 cm), with moderate adherence to the surrounding tissues at the cervicothoracic junction. Finally, we performed a right upper lobectomy and completely excised the tumor without spillage ( Fig. 1B ). The cut section of the tumor showed a well-circumscribed solid and spongy mass, with a homogeneous surface and diffuse microcysts ( Fig. 1C ). Histopathologic examination revealed a well-circumscribed tumorous lesion with a fibrous border and an abrupt interface with the adjacent normal lung ( Fig. 1D ). The surrounding lung was appropriate for gestational age, without remarkable abnormalities, such as glycogen deposition. The cytoplasm of interstitial and epithelial cells showed strong periodic acid-Schiff positivity, digestible by diastase, confirming a histological diagnosis of FLIT.

**Fig. 2:**
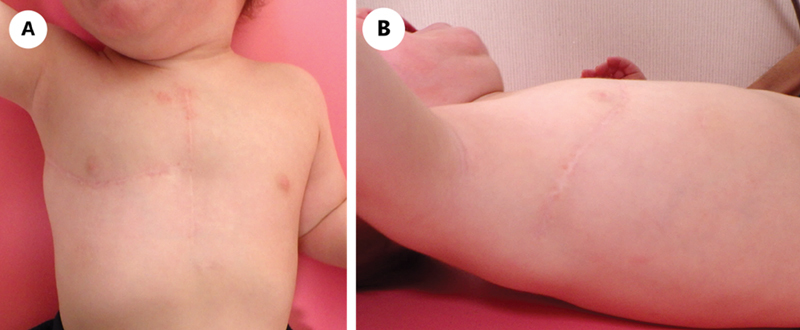
Cosmetic outcomes. Healing of the hemi-clamshell incision is shown at 1-year postsurgery ( **A** : frontal view; **B** : lateral view).

The neonate's postoperative recovery was uneventful. He required minimal ventilator support and was extubated on postoperative day (POD) 2. Oral feeding was resumed on POD 5, and the neonate was discharged on POD 15. Over the subsequent 1-year follow-up, the infant presented no symptoms or observable complaints, with no occurrence of shoulder dysfunction ( Fig. 2 ).

## Discussion

A recent retrospective cohort study of pediatric patients with cervicothoracic tumors reported outcomes of clamshell or trapdoor thoracotomy. 1 However, the use of a hemi-clamshell approach in neonates has not been previously reported, nor has the incidence of complications in pediatric patients been evaluated. 3

The diagnosis of the mass in our patient was difficult at first due to the absence of remarkable features on the first preoperative CT image. This is why we first suspected other primary lung tumor of neonates or a mediastinal tumor. After careful consideration, we diagnosed congenital primary lung tumors. They are rare, and their prevalence is still largely unknown. 4 This makes a radiologic diagnosis very challenging. Preoperative predictions of malignant potential are impossible.

In the absence of a clear diagnosis at the time of surgery, surgical resection of the entire tumor was essential. It was very important to avoid spillage of tumors at the time of resection, especially in tumors like pleuropulmonary blastoma, which were relatively resistant to chemotherapy and radiotherapy. 5 6

In cases with poor overall visualization of the full giant tumor and of the lung hilum, optimal exposure of the operative field is recommended. A posterolateral thoracotomy provides limited visualization and access to the mediastinum. 2 A median sternotomy offers a suitable alternative, particularly for the treatment of cardiac injuries and mediastinal tumors. 2 However, with a median sternotomy, the inferior and posterior regions of the chest cavity, mediastinum, and lungs are less well exposed than with a thoracotomy. The hemi-clamshell approach can provide multiangle views of the pulmonary hilum, posterior–anterior mediastinum, and diaphragm to control hilar and interlobar anatomic dissection, as well as hemorrhage due to an injury of the pulmonary vessels. 7 It is for these reasons that we used a full sternotomy with a lateral thoracotomy by hemi-clamshell approach.

Hemi-clamshell incisions were originally applied for the management of mediastinal tumors or trauma of subclavian vessels. 8

In 1994, Bains et al 9 of the Memorial Sloan-Kettering Cancer Center thoracic unit, followed later by Korst and Burt in 1998, 10 published their results using the hemi-clamshell approach for the resection of the cervicothoracic lesions including superior sulcus tumors. A submammary incision progressed into a partial median sternotomy up to the jugular notch. The chest wall was elevated superiorly and laterally, allowing an excellent exposure of lesions infiltrating the superior mediastinum and lung apex. In addition, this approach can secure the great vessels of the heart by performing full sternotomy. 7 The hemi-clamshell incision can be performed with the patient in the supine position. Specifically, the median sternotomy can be combined with an anterolateral thoracotomy via the fourth or fifth intercostal space and provides an excellent view of the thoracic inlet, including the subclavian vessels, the mediastinum, and the involved chest cavity. 2

Limitations of this report should be acknowledged. The incidence of complications about the hemi-clamshell approach in pediatric patients has not been evaluated. In this approach, we need to be careful about the risk of developing musculoskeletal deformities, including chest wall deformities, scapular anomalies, and scoliosis. The standard thoracotomy approach in neonates may lead to injury of the long thoracic nerve and consequent atrophy of the serratus anterior muscle, resulting in many of the musculoskeletal sequelae associated with thoracotomies. 11 The pathogenic mechanism of median sternotomy in scoliosis is unknown; changes might be subtle but important enough to produce developmental changes in the spine. 12 Previous studies have reported that impaired shoulder girdle function might be related to the effectiveness of postoperative pain control and physiotherapy rather than to the transection of the chest wall muscles. 13 14 Utilizing an incision through the fifth intercostal space for anterior thoracotomy preserves the innervation of the sternal portion of the pectoralis major by the medial pectoral nerve, avoiding postoperative atrophy of this muscle. The medial pectoral nerve is a branch of the medial cord of the brachial plexus, which descends inferiorly on the posterior side of the pectoralis major to innervate the inferior portion of the sternal head for the muscle. 15 16 A muscle-sparing technique, including serratus anterior muscle, minimizes the incidence of musculoskeletal deformities after thoracotomy in children. 11 To avoid morbidity, our approach never resected ribs and carefully performed through the fifth intercostal space to avoid rib fusion and preserve the major chest wall muscles.

As the surgical injury associated with the hemi-clamshell approach may be an etiologic factor in the development of spinal deformity, the use of techniques, such as minimally invasive surgery, which have a low reported rate of spinal and cosmetic problems, might be encouraged.

However, with adequate surgical preparation, this approach should be useful to provide optimal exposure for complete resection of a giant tumor.

In our case, the hemi-clamshell incision provided good exposure to the entire mediastinum and chest cavity, which allowed us to easily perform the right upper lobectomy with complete resection of the mass.

FLIT is a newly identified lung lesion in infants. It is characterized by its morphologic features, including immature airspaces and interstitium, a fibrous capsule, and abundant cytoplasmic glycogen deposition in epithelial and interstitial cells. 17 Histologically, FLIT closely resembles fetal lung tissue at the canalicular stage (20–24 weeks of gestation), pulmonary interstitial glycogenosis (PIG), and congenital cystic adenomatoid malformation/CPAM type 3. In the normal human fetal lung, cytoplasmic glycogen deposition is observed in epithelial cells, particularly type 2 pneumocytes, which are thought to be involved in surfactant synthesis. There are no glycogen granules in interstitial cells of the normal fetal lung at any gestational age. 18 Pulmonary interstitial glycogen granules have only been reported in epithelial and interstitial cells in FLITs and interstitial cells in PIGs. Therefore, the histological diagnosis was FLIT.

## Conclusions

FLITs are rare tumors in neonates that are difficult to diagnose before surgery due to their nonspecific findings and the fact that they are indistinguishable from lung tumors. Our case suggests that a hemi-clamshell approach should be considered in providing optimal exposure for complete resection of a giant tumor with the possibility of malignancy.

## Notes

New Insights and the Importance for the Pediatric Surgeon We believe our case is important considering the rarity of fetal lung interstitial tumors in general and the fact that previously reported cases have not used a hemi-clamshell incision. We believe that our case report will be of interest to the readership of your journal because our experience may help surgeons make the decision to use a hemi-clamshell approach in similar cases of primary pediatric thoracic giant tumors.
